# Cluster-Defined Metabolic Heterogeneity and Longitudinal Effects of Allopurinol and SGLT2 Inhibitors in Hyperuricemic Type 2 Diabetes

**DOI:** 10.3390/medsci14020162

**Published:** 2026-03-24

**Authors:** Roland Fejes, Tamás Jámbor, Andrea Szabó, Szabolcs Péter Tallósy

**Affiliations:** 1 Institute of Surgical Research, Albert Szent-Györgyi Medical School, University of Szeged, 6720 Szeged, Hungary; 2 Department of Internal Medicine, Hódmezővásárhely-Makó Healthcare Center, H-6900 Kórház Street 2, 6900 Makó, Hungary

**Keywords:** hyperuricemia, serum uric acid, type 2 diabetes, metabolic clustering, SGLT2 inhibitors, allopurinol, phenotypic heterogeneity, sex differences

## Abstract

**Background:** Hyperuricemia frequently coexists with type 2 diabetes mellitus (T2DM), contributing to a heterogeneous patient population. While previous analyses compared the overall longitudinal effects of allopurinol and SGLT2 inhibitors in this cohort, it remains unclear whether baseline metabolic heterogeneity modifies treatment response. This study aimed to determine whether data-driven metabolic clustering identifies phenotypic subgroups with prognostic or predictive relevance in hyperuricemic T2DM. **Methods:** In a retrospective cohort of 224 patients with T2DM and hyperuricemia, model-based clustering was applied to age, diabetes duration, body mass index (BMI), serum uric acid (sUA), HbA1c, eGFR, and sex. A sensitivity analysis excluded outliers, yielding 207 patients. Longitudinal trajectories of eGFR and sUA were assessed using linear mixed-effects models and individual slopes. Effect modification by cluster was tested via three-way interactions and analysis of covariance. **Results:** Clustering identified two groups with weak separation: an adipose–metabolic cluster (*n* = 116; exclusively male, BMI 33.1 ± 5.7 kg/m^2^, sUA 478 ± 62 µmol/L) and a lean–metabolic cluster (*n* = 91; exclusively female, BMI 31.3 ± 6.0 kg/m^2^, sUA 426 ± 67 µmol/L). Treatment-agnostic analyses showed no differences in eGFR and sUA slopes or in all-cause mortality across clusters. In both clusters, SGLT2 inhibitors yielded significantly more favourable eGFR slopes than allopurinol, while sUA reductions were comparable across treatments. No significant three-way interactions were detected. **Conclusions:** In this cohort, although baseline metabolic characteristics differ among patients, using the selected baseline variables, no clinically actionable treatment-relevant phenotypes were identified.

## 1. Introduction

Hyperuricemia (HUA) is frequent among patients with type 2 diabetes mellitus (T2DM) and is closely intertwined with obesity, chronic kidney disease (CKD), and cardiovascular (CV) risk [1,2]. Beyond its role as a biochemical abnormality, elevated serum uric acid (sUA) has been linked to metabolic dysfunction and renal microvascular changes [3,4]. Notably, available evidence indicates that HUA is largely epiphenomenal in CV disease and, despite potential nephrotoxic effects at high sUA levels, urate-lowering therapy has not been shown to slow CKD progression [5]. However, hyperuricemic T2DM represents a clinically heterogeneous population, and the extent to which underlying metabolic heterogeneity translates into meaningful phenotypic subgroups remains uncertain [6,7,8].

Traditionally, patient stratification in T2DM has largely relied on markers of disease severity, most prominently glycemic control and the presence or extent of target-organ damage. While clinically pragmatic, such severity-based classification may obscure other biologically relevant dimensions of heterogeneity that could influence long-term outcomes. In response to this limitation, data-driven clustering approaches have gained prominence; however, subsequent large-scale cohort analyses have yielded inconsistent findings regarding the incremental prognostic and predictive value of these subgroups, particularly with respect to differential treatment response. [9,10,11]. This debate is particularly relevant in hyperuricemic T2DM, a condition characterized by intertwined disturbances in metabolism, renal function, and urate handling. In such a setting, baseline metabolic variability may either represent clinically meaningful phenotypes with distinct prognostic or predictive implications or merely reflect gradations along a continuous metabolic spectrum without clear therapeutic consequences.

This uncertainty is particularly relevant given the expanding therapeutic landscape. Sodium–glucose cotransporter 2 (SGLT2) inhibitors have emerged as cornerstone therapies in T2DM, conferring robust kidney and CV protection [12,13,14]. In parallel, xanthine oxidase inhibitors such as allopurinol remain the standard of care for urate lowering but have shown inconsistent effects on kidney outcomes [15,16]. Whether baseline metabolic phenotype or HUA-related traits modify the kidney or urate-lowering effects of these treatments is largely unknown [17]. Addressing this question is central to determining whether precision-medicine approaches based on metabolic phenotyping are justified in hyperuricemic T2DM.

While our previous analysis in this cohort compared the overall longitudinal effects of allopurinol and SGLT2 inhibitors, it did not address whether baseline metabolic heterogeneity modifies treatment response [18]. The present study therefore extends prior work by applying an unsupervised clustering framework to determine whether phenotype-guided therapeutic stratification is supported by differential longitudinal effects on kidney function and sUA. Accordingly, we applied model-based clustering to baseline metabolic and clinical variables to identify putative phenotypes and evaluated both their prognostic relevance and their potential to modify longitudinal treatment effects on estimated glomerular filtration rate (eGFR), serum uric acid, and all-cause mortality. By integrating unsupervised phenotyping with formal interaction testing, this study sought to distinguish descriptive metabolic heterogeneity from clinically actionable phenotypic stratification in hyperuricemic T2DM.

## 2. Materials and Methods

### 2.1. Study Population and Data Sources

The present study is a secondary analysis of a previously established retrospective cohort of patients with T2DM and HUA, as initially described in detail elsewhere [18]. No modifications were made to the original dataset or data collection procedures. Given the design and sample size, no a priori power calculation was performed for interaction testing; therefore, analyses of effect modification should be interpreted as exploratory.

Briefly, the cohort comprised Caucasian adults (over 18 years) attending an outpatient diabetes clinic at a secondary care hospital in Hungary. Patients were included if treatment with either an SGLT2 inhibitor (empagliflozin 10 mg/day or dapagliflozin 10 mg/day) or allopurinol (100 mg/day) was initiated between 1 January 2017 and 1 January 2020. Patient selection, including inclusion/exclusion criteria and the resulting case numbers, is detailed in Supplementary Material S1. Hyperuricemia was defined according to sex-specific thresholds (>416 µmol/L in men; >357 µmol/L in women) [19]. A comprehensive table of baseline clinical characteristics stratified by treatment is provided in Supplementary Table S1.

Exclusion criteria were identical to those in the prior report: sub-threshold baseline sUA, concomitant use of allopurinol and SGLT2 inhibitors, dose escalation during follow-up, or incomplete records. The index visit at treatment initiation served as baseline (BV), with follow-up data collected at approximately 12, 24, and 36 months.

The study complied with the Declaration of Helsinki (2008 revision). Ethical approval was granted by the Institutional Review Board of Hódmezővásárhely-Makó Healthcare Center and the Hungarian National Public Health Center Institutional Committee of Science and Research Ethics (NNGYK/65897-2/2025; approved 28 October 2025). All data were fully anonymized, and individual consent was waived owing to the retrospective design.

### 2.2. Cluster Analysis and Validation

Cluster analysis was performed via the machine-learning module of JASP software (v.0.95.4, University of Amsterdam, Amsterdam, The Netherlands). The analysis was restricted to baseline variables (age, diabetes duration, BMI, sUA, HbA1c, eGFR, and sex). Sex was included as a clinically relevant indicator of metabolic heterogeneity; however, given that binary variables may influence Gaussian-mixture–based solutions, we performed a sensitivity analysis excluding sex to assess robustness of the inferred cluster structure. Furthermore, longitudinal parameters were excluded as they reflect disease progression or treatment effects rather than baseline phenotypic structure.

Continuous clustering variables were entered on their original, clinically interpretable scales without log-transformation. To reduce the potential influence of extreme values and long-tailed distributions, a predefined z-score–based outlier filter (|z| ≥ 3 for any continuous clustering variable) was applied before model-based clustering. Extreme values tended to generate small, unstable clusters with limited clinical interpretability; accordingly, the filtered sensitivity cohort was used for primary cluster derivation, whereas the full cohort was preserved for descriptive reporting to ensure transparency. Sex was included as a binary variable without transformation. Cluster solutions with two to four clusters (K = 2–4) were evaluated. Model selection was guided by cluster size, silhouette scores, visual separation assessed by t-distributed stochastic neighbor embedding (t-SNE), and overall clinical interpretability.

Cluster characteristics were summarized using cluster-specific mean standardized values, which informed post hoc phenotype labeling. Phenotypes were named to reflect relative metabolic features within the hyperuricemic cohort, avoiding absolute clinical categorization.

### 2.3. Further Statistical Analysis

Normality was assessed with the Shapiro–Wilk test. Normally distributed variables are presented as mean ± SD, while skewed non-normal variables are displayed as median (25th–75th percentile). Categorical variables are summarized as *n* (%), with between-group comparisons by Fisher’s exact test. Continuous baseline variables were compared using independent-samples *t* tests.

Longitudinal changes in eGFR and sUA were analyzed using linear mixed-effects models with random intercepts and slopes to account for within-subject correlation. Fixed effects included time, treatment group, phenotype, and their interactions. Results are reported as estimated slopes with 95% confidence intervals (CI). To assess whether longitudinal treatment effects differed by phenotype, a three-way interaction term (time × treatment × phenotype) was included, with significance evaluated using Wald tests. To complement mixed-effects modeling and facilitate phenotype-level comparisons, individual annual slopes of eGFR and sUA were additionally derived using participant-level linear regression of longitudinal measurements, requiring a minimum of three time points per individual.

Multivariable linear regression models were then fitted with eGFR slope and sUA slope as continuous dependent variables. Treatment group and phenotype were included as categorical predictors, while baseline eGFR and baseline sUA were included as continuous covariates. Allopurinol was specified as the reference treatment category. To formally assess effect modification by phenotype, analysis of covariance models were constructed, including treatment, phenotype, baseline value of the respective outcome, and a treatment × phenotype interaction term. Type III sums of squares were used to evaluate main and interaction effects. In the absence of a statistically significant interaction, treatment effects were interpreted as consistent across phenotypes.

All available longitudinal measurements were included in the mixed-effects models irrespective of the number of follow-up visits, as this approach accommodates unbalanced data; individual slope analyses were restricted to participants with ≥3 measurements to ensure stable trajectory estimation.

All-cause mortality was analyzed as a time-to-event outcome using Kaplan–Meier methods with log-rank testing, and unadjusted Cox proportional hazards models were fitted to estimate hazard ratios.

All analyses were two-sided, and a *p* value < 0.05 was considered statistically significant.

## 3. Results

### 3.1. Defining Clusters

Stepwise exploration and refinement of the clustering structure are presented in Supplementary Material S2. A total of 224 patients with complete baseline data were included, allowing clustering without imputation. Seventeen patients (7.5%) met the predefined criterion for extreme values (absolute z-score ≥ 3 for any clustering variable) and were temporarily excluded, yielding a sensitivity cohort of 207 patients.

**Figure 1:**
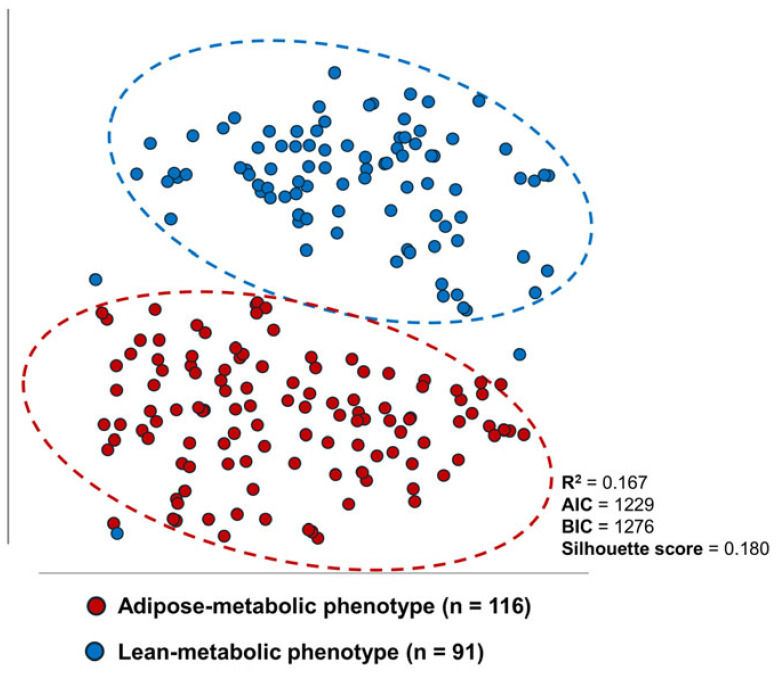
t-SNE visualization of model-based clustering results. Two distinct patient clusters are shown, corresponding to an adipose-metabolic cluster with male predominance (red dataset) and a lean–metabolic cluster with female predominance (blue dataset). Although visually distinguishable, overall separation was weak (overall silhouette score = 0.180), indicating substantial overlap between groups. Axes represent t-SNE dimensions and have no direct clinical interpretation.

Model-based clustering in the sensitivity cohort identified a two-cluster (K = 2) solution as the most stable and clinically plausible structure, with cluster sizes of 116 and 91 patients, respectively. Visualization using t-distributed stochastic neighbor embedding (t-SNE) showed two patient groups with visually discernible separation and limited overlap (Figure 1).

Cluster separation was primarily driven by BMI, sUA, and sex. One cluster was characterized by higher BMI and sUA values, with male predominance. In contrast, the other showed lower BMI and sUA values with female predominance (cluster-specific standardized means are shown in Supplementary Table S2). Other baseline variables contributed minimally to cluster separation. Based on these characteristics, the two groups are hereafter referred to as an adipose–metabolic cluster and a lean–metabolic cluster.

As part of the sensitivity analysis, solutions with K = 3 and K = 4 were also examined. In the K = 3 analysis, one cluster exhibited overlap, while the K = 4 solution resulted in a small cluster with deteriorating separation metrics (Table 1; Supplementary Materials S3 and S4).

### 3.2. Validation of Cluster Analysis

The K = 2 cluster solution yielded an *R*^2^ of 0.167, an overall silhouette score of 0.180, with similar cluster-specific silhouette values (0.178 and 0.183) (Table 1). For BMI, the pooled within-cluster standard deviation was 5.80, compared with a total cohort standard deviation of 5.86, resulting in a within-to-total standard deviation ratio of 0.99. For sUA, the pooled within-cluster standard deviation was 64.3, compared with a total cohort standard deviation of 69.2, corresponding to a ratio of 0.93. Collectively, these validation metrics suggest weak separation and considerable within-cluster heterogeneity, consistent with limited structural distinctiveness. Additional validation analyses are presented in Supplementary Material S5.

To address the complete sex separation observed in the primary clustering solution, we performed a sensitivity analysis excluding sex from the clustering variables. Model-based clustering without sex again identified a two-cluster solution with similarly weak separation, low silhouette values, and substantial within-cluster heterogeneity, indicating no emergence of distinct metabolic phenotypes (cluster sizes *n* = 82 and *n* = 125; overall silhouette score = 0.120; *R*^2^ = 0.117). Detailed results of this sex-excluded clustering analysis are provided in Supplementary Material S6.

### 3.3. Baseline Clinical Characteristics According to Clusters

Baseline clinical characteristics stratified by cluster-derived groups are shown in Table 2. Patients classified into the adipose–metabolic cluster exhibited significantly higher BMI and sUA levels compared with those in the lean–metabolic cluster. In contrast, age, duration of diabetes, HbA1c, and eGFR did not differ between the two clusters.

Sex distribution showed complete separation between clusters: all patients in the adipose–metabolic cluster were male, whereas all patients in the lean–metabolic cluster were female.

### 3.4. Treatment-Agnostic Longitudinal Outcomes According to Cluster-Derived Clusters

**Figure 2:**
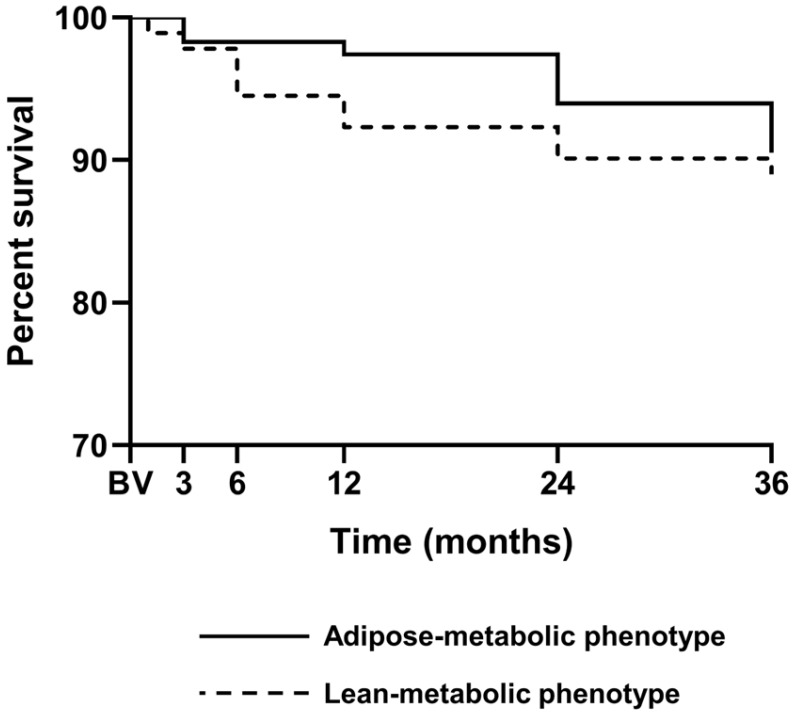
Kaplan–Meier analysis of all-cause mortality by cluster. Changes are expressed as percentages of the BV case numbers. The adipose–metabolic cluster is shown with a solid line, and the lean–metabolic cluster with a dashed line.

Analyses of individual longitudinal slopes were restricted to patients with at least three available measurements. When analyzed independently of treatment allocation, cluster-defined groups showed no statistically significant differences in longitudinal trajectories of sUA and eGFR (*p* = 0.785 and *p* = 0.150, respectively; Table 3). During follow-up, all-cause mortality occurred in 12 patients (5.8%) in the adipose–metabolic cluster and in 11 patients (5.3%) in the lean–metabolic cluster. Kaplan–Meier survival curves did not differ between clusters (*p* = 0.676; Figure 2). Consistently, unadjusted Cox proportional hazards analysis showed no significant association between cluster assignment and all-cause mortality (hazard ratio 0.83, 95% CI 0.35–1.98).

### 3.5. Cluster-Specific Longitudinal Treatment Effects on eGFR and sUA

Baseline treatment allocation did not differ between cluster-defined groups (Supplementary Material S7). Cluster-stratified longitudinal treatment effects are summarized in Table 4.

Longitudinal changes in eGFR: In both the adipose–metabolic and lean–metabolic clusters, patients treated with allopurinol experienced a significant annual decline in eGFR (*p* < 0.001 for both). In contrast, treatment with SGLT2 inhibitors was associated with a markedly attenuated decrease in eGFR, resulting in significantly more favorable slopes compared with allopurinol in both clusters (empagliflozin and dapagliflozin vs. allopurinol, all *p* < 0.001). Within each cluster, the time × treatment interaction was statistically significant (*p* < 0.001 in both clusters), indicating differential eGFR trajectories across treatment groups.

Longitudinal changes in sUA: Across both clusters, all treatments were associated with reductions in sUA levels over time. In the adipose–metabolic cluster, the annual decline in sUA did not differ significantly between allopurinol and either empagliflozin or dapagliflozin (*p* = 0.528 and *p* = 0.544, respectively), and no significant time × treatment interaction was observed (*p* = 0.778). Similarly, in the lean–metabolic cluster, sUA declined across all treatment groups, with no statistically significant differences in slopes between SGLT2 inhibitors and allopurinol (all *p* > 0.20). The cluster-stratified time × treatment interaction for sUA was not statistically significant (*p* = 0.425).

Three-way interaction analysis: In the global models including the time × treatment × cluster interaction, no statistically significant three-way interaction was detected for either eGFR (*p* = 0.188) or sUA (*p* = 0.433).

### 3.6. Determinants of eGFR and Serum Uric Acid Slopes

In multivariable models adjusted for baseline values, treatment was strongly associated with the annual slope of eGFR (*p* < 0.001, Table 5). Compared with allopurinol, dapagliflozin was associated with a significantly smaller decline in eGFR (*p* < 0.001), whereas empagliflozin did not differ significantly from allopurinol (*p* = 0.921). Cluster assignment was independently associated with eGFR slope (*p* = 0.032), and baseline eGFR remained a significant covariate (*p* = 0.042). No statistically significant treatment × cluster interaction was observed for eGFR slope (*p* = 0.611).

For sUA slope, no overall treatment effect was detected (*p* = 0.407), and neither empagliflozin nor dapagliflozin differed significantly from allopurinol. Baseline sUA was the strongest determinant of sUA slope (*p* < 0.001). Cluster assignment was not independently associated with sUA slope after adjustment (*p* = 0.091). The treatment × cluster interaction was not statistically significant (*p* = 0.052).

## 4. Discussion

Hyperuricemia and T2DM frequently coexist, share a multifactorial pathogenesis, and serve as common etiological factors for several comorbid conditions, including CKD and atherosclerotic CV disease [20,21]. This shared pathophysiology results in a remarkably heterogeneous patient population in which metabolic traits—such as obesity, elevated sUA, chronic inflammation, and sex-related differences—contribute substantially to variability in disease presentation, progression, and potentially treatment response [22,23]. Clinically, this underscores the need to clarify whether baseline metabolic differences should influence therapeutic decision-making or whether treatments can be applied broadly across patients. Allopurinol remains a dominant urate-lowering therapy; however, the uricosuric effects of SGLT2 inhibitors—observed alongside their established multidomain cardiovascular and kidney benefits—have attracted increasing attention in recent years [24,25,26]. The primary objective of this study was not to demonstrate the superiority of one treatment over another, but rather to examine whether baseline metabolic clustering modifies longitudinal trajectories of eGFR or sUA in patients treated with either allopurinol or SGLT2 inhibitors, such as empagliflozin or dapagliflozin. This question was addressed through a secondary analysis of the dataset used in our previous study, with the aim of exploring potential factors that might influence the findings reported there [18].

Model-based clustering identified two groups that differed primarily along dimensions of BMI, sUA, and sex; however, validation metrics indicated weak separation. Statistically, this suggests that the groups do not represent sharply demarcated, discrete phenotypes but rather the extremes of a continuous metabolic axis along which patients are distributed with gradual transitions and considerable overlap. Although silhouette values below 0.25 are generally interpreted as indicating weak or absent cluster structure, such values are not uncommon in real-world clinical datasets characterized by substantial biological and phenotypic heterogeneity. Clinically, this finding suggests that hyperuricemic T2DM may represent a heterogeneous condition rather than a homogeneous entity, with patients potentially better viewed along a continuum rather than as rigidly defined categories. Clustering here serves as a descriptive tool to highlight dominant sources of variation (e.g., the relative contributions of obesity and hyperuricemia). Yet, it does not warrant the introduction of new diagnostic or therapeutic subgroups.

Particularly noteworthy was the complete separation of clusters by sex, with the adipose–metabolic cluster comprising exclusively men and the lean–metabolic cluster exclusively women. These findings underscore sex as a dominant contributor to baseline metabolic heterogeneity in hyperuricemic T2DM, consistent with well-established biological differences in body fat distribution and renal urate handling (e.g., androgen-driven visceral adiposity and reduced renal urate clearance in men, and estrogen-mediated protective effects on urate handling in premenopausal women) [27,28,29,30,31]. To determine whether this pronounced sex effect artificially drove the observed clustering structure, we performed a dedicated sensitivity analysis excluding sex from the clustering variables; this analysis yielded similarly weak cluster separation and did not reveal more distinct or clinically interpretable metabolic phenotypes. Importantly, despite these marked sex-related baseline differences, no sex-specific treatment strategies emerged as clinically necessary.

The complete sex separation, therefore, provided a stringent test of effect modification rather than a methodological limitation. Despite such pronounced sex-related baseline differences, no significant time × treatment × cluster interactions were observed for either eGFR or sUA, indicating that treatment effects remained consistent across the metabolic continuum even in the presence of strong sex-related influences. Consequently, within the constraints of this retrospective cohort and the selected baseline variables, we did not observe clinically meaningful sex-specific or phenotype-dependent differences in treatment response, supporting the application of uniform therapeutic strategies across sexes and reducing the risk of sex-based therapeutic stratification.

Treatment-agnostic analyses revealed no differences between clusters in sUA or eGFR trajectories or in all-cause mortality. In multivariable models, cluster membership showed only a modest independent association with eGFR decline, but not with sUA trajectories, suggesting limited prognostic relevance. Importantly, this modest prognostic association did not translate into differential treatment response. Thus, while cluster assignment may convey some information regarding baseline renal risk, it does not appear to predict differential benefit from allopurinol or SGLT2 inhibitors. Baseline position along this metabolic axis adds little incremental value to routine risk assessment beyond established models; although obesity-related factors may contribute to CKD progression, their impact is not dominant and does not justify additional monitoring or subgroup-specific interventions. The core question of effect modification was addressed using cluster-stratified longitudinal models and formal interaction testing. In both clusters, the SGLT2 inhibitors empagliflozin and dapagliflozin were associated with significantly more favourable eGFR slopes compared with allopurinol, whereas sUA reductions were comparable across all three treatments. The absence of higher-order interactions provides consistent statistical evidence that baseline metabolic heterogeneity does not modify treatment effects. These findings reinforce the robust renoprotective role of SGLT2 inhibitors in hyperuricemic T2DM—an effect magnitude aligned with large randomized controlled trials (e.g., DAPA-CKD, EMPA-KIDNEY)—while allopurinol appears effective primarily as urate-lowering therapy without comparable renal protection [32,33,34]. SGLT2 inhibitors thus emerge as multifunctional agents that address glycemic control, renal preservation, and urate reduction through alternative mechanisms, independent of baseline metabolic phenotype [35,36,37]. Multivariable analyses further clarified the distinction between prognostic association and effect modification: cluster membership was associated with modest renal risk but did not appear to meaningfully modify treatment responses within this cohort. This represents an essential statistical and clinical lesson—variables may be prognostic without being predictive of differential treatment benefit.

As allopurinol was administered at a fixed starting dose of 100 mg/day without dose titration, the study design may underestimate its maximal urate-lowering capacity and any potential pleiotropic effects that might emerge at higher doses. In routine clinical practice, dose escalation of allopurinol is frequently not pursued to the maximally recommended or target dose, and treatment adherence may also vary; therefore, the fixed-dose approach used in this cohort may partly reflect real-world prescribing patterns while potentially underrepresenting the full therapeutic potential of titrated therapy.

In summary, in this cohort, baseline metabolic differences among patients with hyperuricemic T2DM appeared to reflect gradual variation along a continuous metabolic axis and did not identify clearly actionable treatment-relevant phenotypes. Although these differences may influence overall risk, they do not result in clinically relevant differences in treatment response, supporting the broad application of evidence-based therapies without additional phenotypic subdivision.

The present study has several limitations. As a single-center, retrospective cohort in a predominantly Caucasian population, generalizability is limited, and causal inferences are precluded due to potential confounding by indication and residual confounding despite adjustments. Given the retrospective design and modest sample size, this secondary analysis should be regarded as hypothesis-generating. The study was not specifically powered to detect modest treatment × cluster interactions; therefore, absence of statistical interaction should be interpreted cautiously, as the possibility of Type II error due to limited statistical power cannot be fully excluded. Larger prospective studies are warranted to more robustly assess potential differential treatment responses. Furthermore, continuous variables were entered into the Gaussian mixture model on their original clinical scales without distributional transformation. Although extreme observations were addressed through z-score–based filtering and cluster separation was not driven by a single skewed variable, the potential influence of non-normal distributions on mixture-based clustering cannot be completely excluded. Follow-up was restricted to approximately 36 months, with low event rates. Allopurinol was evaluated at a fixed 100 mg/day starting dose without titration; although guideline-concordant in this asymptomatic cohort, this limits extrapolation to settings requiring higher doses. The lack of urinary albumin-to-creatinine ratio data prevented full KDIGO G–A staging and may have obscured albuminuria-specific effects. Clustering relied on a limited set of seven baseline variables, yielding weakly separated groups along a continuous axis; inclusion of additional markers (e.g., inflammation, insulin resistance) might have refined phenotypic structure [38]. Although sex exerted a strong influence on baseline clustering, this was addressed through a prespecified sensitivity analysis excluding sex from the clustering variables, which yielded similarly weak separation and did not reveal more distinct phenotypes. Nevertheless, as sex was treated as a binary indicator within a Gaussian mixture framework, alternative mixed-data clustering approaches may further refine phenotypic characterization in larger cohorts. Finally, eGFR and sUA measurements were obtained only at scheduled visits, potentially influenced by unmeasured factors (e.g., diet, diuretics, intercurrent illness).

## 5. Conclusions

In this retrospective cohort of patients with hyperuricemic T2DM, data-driven metabolic clustering identified baseline differences mainly related to BMI, sUA, and sex, but these clusters showed weak separation. Although cluster membership was associated with some baseline metabolic variation and showed modest prognostic association with renal trajectories, it did not demonstrate predictive value with respect to differential treatment response. Across both clusters, SGLT2 inhibitors were associated with more favorable eGFR trajectories than allopurinol, while reductions in sUA were comparable between treatments. Overall, these findings suggest that in hyperuricemic T2DM, baseline metabolic heterogeneity—at least as captured by the selected variables—may carry limited prognostic information but does not support clinically actionable phenotype-guided treatment stratification.

## Figures and Tables

**Table 1:** Model comparison metrics for model-based clustering solutions (K = 2–4) in the outlier-excluded cohort (*n* = 207). Models were evaluated using *R^2^* (proportion of explained variance), Akaike Information Criterion (AIC), Bayesian Information Criterion (BIC), and overall silhouette score.

Clusters (K)	*R* ^2^	AIC	BIC	Silhouette Score
2	0.167	1229	1276	0.180
3	0.118	1291	1361	0.030
4	0.159	1258	1351	0.010

**Table 2:** Baseline clinical characteristics according to clusters. Data are presented as medians with 25th–75th percentiles. Sex is presented as counts and percentages; no statistical test was applied due to complete separation between clusters. Abbreviations: body mass index, BMI; serum uric acid, sUA; glycated Hb, HbA1c; estimated glomerular filtration rate, eGFR.

Variable	Adipose–Metabolic (*n* = 116)	Lean–Metabolic (*n* = 91)	*p* Value
Age (years)	63 (55; 68)	63 (55.5; 68)	0.860
Diabetes duration (years)	10 (5; 15)	10 (5; 15)	0.617
BMI (kg/m^2^)	32.6 (29.3; 36.7)	30.6 (27.6; 34.8)	0.023
sUA (µmol/L)	466 (444; 511)	403 (385; 436)	<0.001
HbA1c (%)	7.9 (7.6; 8.7)	8.1 (7.7; 8.6)	0.436
eGFR (mL/min/1.73 m^2^)	74.5 (47.75; 90)	74 (51; 90)	0.956
Male sex (*n*, %)	116 (100%)	0 (0%)	—

**Table 3:** Treatment-agnostic comparison of longitudinal slopes according to clusters. Values are shown as mean ± SD. Abbreviations: serum uric acid, sUA; estimated glomerular filtration rate, eGFR.

Outcome	Adipose–Metabolic Cluster (*n* = 85)	Lean–Metabolic Cluster (*n* = 62)	*p* Value
sUA slope (µmol/L per year)	−29.3 ± 26.2	−26.0 ± 19.4	0.785
eGFR slope (mL/min/1.73 m^2^ per year)	−1.55 ± 3.75	−0.23 ± 4.07	0.150

**Table 4:** Annual eGFR and sUA slopes. Results are presented as yearly slopes with 95% CIs. Allopurinol served as the reference treatment; the Δ slope vs allopurinol column indicates the difference in slope relative to allopurinol. The *p*-values refer to pairwise comparisons versus allopurinol. Global time × treatment interaction *p*-values are reported separately for each cluster to test whether the rate of change over time differs between treatment groups within the given cluster. Abbreviations: serum uric acid, sUA; estimated glomerular filtration rate, eGFR.

eGFR Slope (mL/min/1.73 m^2^ per Year)				
Cluster	Treatment	Slope per Year (95% CI)	Δ Slope vs. Allopurinol (95% CI)	*P* (vs. Allopurinol)
Adipose–metabolic	Allopurinol	−3.67 (−4.61 to −2.73)	Reference	-
Adipose–metabolic	Empagliflozin	−0.48 (−1.41 to 0.44)	3.19 (1.87 to 4.51)	<0.001
Adipose–metabolic	Dapagliflozin	−0.68 (−1.58 to 0.22)	2.99 (1.69 to 4.30)	<0.001
Lean–metabolic	Allopurinol	−3.12 (−4.28 to −1.97)	Reference	-
Lean–metabolic	Empagliflozin	1.02 (−0.58 to 2.62)	4.15 (2.55 to 5.75)	<0.001
Lean–metabolic	Dapagliflozin	1.81 (0.44 to 3.18)	4.94 (3.26 to 6.62)	<0.001
Global time × treatment interaction *p*-value (cluster-stratified):				
Adipose–metabolic: *p* < 0.001				
Lean–metabolic: *p* < 0.001				
**sUA slope (µmol/L per year)**				
**Cluster**	**Treatment**	**Slope per year (95% CI)**	**Δ slope vs. allopurinol (95% CI)**	***P*** **(vs. allopurinol)**
Adipose–metabolic	Allopurinol	−32.66 (−40.58 to −24.74)	Reference	-
Adipose–metabolic	Empagliflozin	−28.46 (−37.55 to −19.38)	4.20 (−8.83 to 17.22)	0.528
Adipose–metabolic	Dapagliflozin	−28.71 (−37.39 to −20.03)	3.95 (−8.81 to 16.71)	0.544
Lean–metabolic	Allopurinol	−23.54 (−32.78 to −14.29)	Reference	-
Lean–metabolic	Empagliflozin	−25.66 (−34.49 to −16.83)	−2.12 (−14.89 to 10.65)	0.745
Lean–metabolic	Dapagliflozin	−32.15 (−41.87 to −22.43)	−8.62 (−22.00 to 4.77)	0.207
Global time × treatment interaction *p*-value (cluster-stratified):				
Adipose–metabolic: *p* = 0.778				
Lean–metabolic: *p* = 0.425				

**Table 5:** Determinants of eGFR and serum uric acid (sUA) slopes assessed by multivariable linear regression and ANCOVA. Annual eGFR and sUA slopes were analyzed as continuous outcomes. β coefficients and 95% CIs are derived from multivariable linear regression models adjusted for baseline eGFR or baseline sUA, respectively. Results are derived from multivariable regression models using individual patient slopes (≥3 measurements) and adjusted for baseline values and cluster assignment; differences from mixed-effects estimates (Table 4) reflect differences in modelling approach and covariate structure. Overall effects and interaction *p* values are derived from ANCOVA. Allopurinol was used as the reference treatment. Treatment × cluster interaction terms were included to assess effect modification by cluster; a non-significant interaction indicates the absence of cluster-dependent treatment effects. Abbreviations: serum uric acid, sUA; estimated glomerular filtration rate, eGFR.

Determinants of eGFR Slope: Multivariable Linear Regression and ANCOVA			
Effect	*β* (95% CI)	*p* Value	Interpretation
Treatment	-	<0.001	Strong overall treatment effect
Empagliflozin vs. allopurinol	−0.07 (−1.47 to 1.33)	0.921	No difference vs. reference
Dapagliflozin vs. allopurinol	3.58 (2.17 to 4.98)	<0.001	Smaller decline vs. reference
Between clusters	1.27 (0.12 to 2.42)	0.032	Independent prognostic effect
Baseline eGFR	−0.029 (−0.057 to −0.002)	0.042	Significant covariate
Treatment × Cluster	-	0.611	No effect modification
Determinants of sUA slope: multivariable linear regression and ANCOVA			
Effect	*β* (95% CI)	*p* value	Interpretation
Treatment	-	0.407	No overall treatment effect
Empagliflozin vs. allopurinol	2.00 (−6.26 to 10.26)	0.633	No difference vs. reference
Dapagliflozin vs. allopurinol	−4.80 (−13.19 to 3.60)	0.261	No difference vs. reference
Between clusters	−6.23 (−13.67 to 1.20)	0.091	Non-significant trend
Baseline sUA	−0.187 (−0.242 to −0.131)	<0.001	Strong baseline dependence
Treatment × Cluster	-	0.052	Borderline interaction

## Data Availability

Data are available from the corresponding authors upon reasonable request; access is restricted due to ethical approval requirements.

## Supplementary Materials

The following supporting information can be downloaded at: https://www.mdpi.com/article/10.3390/medsci14020162/s1. Table S1. Clinicopathologic characteristics of patients enrolled in the study. Supplementary Material S1. Schematic flowchart showing the exclusion criteria used for patient selection. Supplementary Material S2. Stepwise exploration and refinement of the clustering structure. Table S2. Cluster-specific mean standardized values of K = 2 solution. Supplementary Material S3. Evaluation of the three-cluster solution in the filtered cohorts. Supplementary Material S4. Evaluation of the four-cluster solution in the filtered cohorts. Supplementary Material S5. Detailed validation of cluster analysis. Supplementary Material S6. Sensitivity analysis excluding sex from clustering variables. Supplementary Material S7. Baseline treatment allocation according to cluster-derived phenotypes.
