# Supplementary material for: Cluster-Defined Metabolic Heterogeneity and Longitudinal Effects of Allopurinol and SGLT2 Inhibitors in Hyperuricemic Type 2 Diabetes
Source: Med Sci (Basel). 2026 Mar 24;14(2):162. doi: 10.3390/medsci14020162 (PMC13108030; doi:10.3390/medsci14020162)
Supplement: Supplementary file 1 [file medsci-14-00162-s001.zip › medsci-4179873-supplementary.pdf]

Supplementary Materials: Cluster-Defined Metabolic Heterogeneity and Longitudinal Effects of Allopurinol and SGLT2 Inhibitors in Hyperuricemic Type 2 Diabetes

Roland Fejes , Tamás Jámbor , Andrea Szabó and Szabolcs Péter Tallósy

Supplementary Table S1. Clinicopathologic characteristics of patients enrolled in the study. Data are presented as medians with 25th–75th percentiles. *P* values of <0.05 were considered to indicate statistical significance. **p* < 0.05 vs. dapagliflozin.

	Dapagliflozin (n = 78)	Empagliflozin (n = 70)	Allopurinol (n = 66)	*p* value
**Age (years)**	63.0 (54.8; 68.3)	61.5 (54.0; 68.3)	64.0 (57.0; 68.3)	0.717
**Diabetes duration (years)**	10.0 (4.0; 14.3)	10.0 (6.0; 15.0)	10.5 (4.8; 18.3)	0.608
**Body weight (kg)**	101.0 (85.8; 115.0)	96.0 (83.0; 114.3)	104.0 (89.8; 113.5)	0.218
**Body mass index (kg/m^2)**	30.5 (28.0; 35.5)	31.8 (27.5; 36.1)	32.7 (30.1; 36.8)	0.053
**Fasting plasma glucose (mmol/L)**	9.3 (7.2; 11.2)	9.3 (7.9; 12.2)	8.9 (8.5; 9.5)	0.722
**HbA_{1c} (%)**	**8.5 (7.9; 9.0)**	8.2 (7.6; 8.7)	**7.8 (7.5; 8.1)***	***<0.001**
**Total cholesterol (mmol/L)**	5.2 (4.4; 6.0)	5.5 (4.4; 6.1)	5.8 (4.6; 6.3)	0.188
**HDL-cholesterol (mmol/L)**	1.3 (1.1; 1.6)	1.3 (1.1; 1.7)	1.4 (1.1; 1.6)	0.495
**LDL-cholesterol (mmol/L)**	3.4 (2.6; 3.9)	3.4 (2.7; 4.0)	4.0 (2.8; 4.5)	0.061
**Serum uric acid (μmol/L)**	450 (418; 501)	452 (401; 497)	438 (388; 510)	0.813
**Estimated glomerular filtration rate (mL/min/1.73 m^2)**	68.0 (45.0; 90.0)	77.2 (58.0; 90.0)	75.0 (55.0; 90.0)	0.300
**Left ventricular ejection fraction (%)**	56.0 (50.0; 60.0)	55.0 (45.0; 60.0)	60.0 (40.0; 60.0)	0.602
**Insulin usage (n, %)**				
**No insulin usage**	32 (41.0%)	29 (41.4%)	39 (59.1%)	0.063
**Basal insulin supported oral therapy**	7 (8.97%)	7 (10.0%)	3 (4.54%)	0.088
**Fixed-ratio combination therapy**	1 (1.28%)	4 (5.71%)	0 (0.00%)	0.079
**Multiple-dose injection therapy**	38 (48.7%)	30 (42.9%)	24 (36.4%)	0.333
**Non-insulin antidiabetic drug usage (n, %)**				
**Metformin**	59 (75.6%)	51 (72.9%)	49 (74.2%)	0.923
**Sulfonylurea**	19 (24.4%)	18 (25.7%)	20 (30.3%)	0.717
**Dipeptidyl peptidase-4 inhibitor**	11 (14.1%)	6 (8.57%)	14 (21.2%)	0.111
**Glucagon-like peptide-1 receptor agonists**	1 (1.28%)	1 (1.43%)	5 (7.56%)	0.097
**Comorbidities (n, %)**				
**Hypertension**	44 (56.4%)	47 (67.1%)	48 (72.7%)	0.113
**Chronic coronary artery disease**	21 (26.9%)	15 (21.4%)	20 (30.3%)	0.651
**Diabetic retinopathy**	14 (17.9%)	11 (15.7%)	15 (22.7%)	0.574
**Peripheric sensory neuropathy**	25 (32.1%)	32 (45.7%)	29 (43.9%)	0.310
**Chronic kidney disease**	31 (39.7%)	21 (30.0%)	33 (50.0%)	0.058
**Active malignancy**	2 (2.56%)	2 (2.86%)	1 (1.51%)	1.000

Supplementary Material S1. Schematic flowchart showing the exclusion criteria used for patient selection.

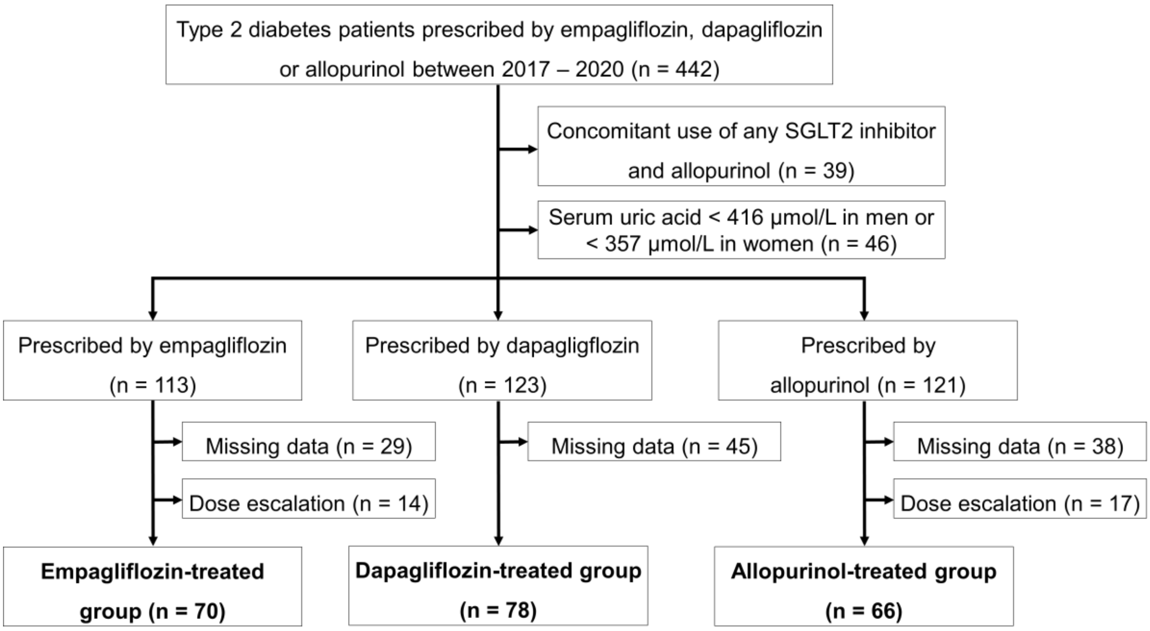

Supplementary Material S2. Stepwise exploration and refinement of the clustering structure.

Cluster analysis was performed using the machine learning module of JASP software (v0.95.4; University of Amsterdam, Amsterdam, The Netherlands). As a first, exploratory step, hierarchical clustering was applied using Ward's linkage method, based on Euclidean distances calculated from standardized input variables. Model selection according to the Bayesian Information Criterion (BIC) suggested a K = 7 solution in the unfiltered population (n = 218). Although this solution resulted in a relatively high overall R^2 value (0.586) and an acceptable silhouette index (0.180), examination of cluster sizes revealed significant inequality; however, the number of individuals in each cluster varied between 5 and 68, with several clusters accounting for less than 10% of the total sample. These small clusters explained only a minimal fraction of the heterogeneity within the clusters (explained proportion: 0.048–0.051), and were therefore not suitable for representing stable or clinically interpretable phenotypes.

Subsequently, we applied model-based clustering using Gaussian mixture models. In the unfiltered population, we examined the K = 3 and K = 4 cluster solutions. In the case of the K = 4 solution, the cluster sizes were 79, 91, 38 and 6 individuals. Although the smallest cluster showed a high silhouette value (0.420) and was characterized by extreme z-scores for several variables (e.g., BMI 3.93; sUA 3.71; HbA1c 4.30). The K = 3 solution showed a similar result. These patterns suggested that solutions with higher cluster counts served primarily to isolate outliers rather than uncover additional, biologically relevant structures.

Based on the results above, extreme observations were thought to disproportionately influence cluster formation; therefore, we performed a predefined sensitivity analysis to examine the robustness of the cluster structure. For each continuous clustering variable, z-score values were calculated according to the following formula:

$$z_{ij} = \frac{x_{ij} - \mu_j}{\sigma_j}$$

where x_{ij}denotes the observed value of variable j for individual i, and $\mu_j$and σ_j represent the mean and standard deviation of variable j in the cohort.

Patients were considered extreme outliers if their absolute z-score for any variable was ≥3. This criterion identified 17 patients, corresponding to approximately 7.5% of the entire cohort. These patients were temporarily excluded using a global filter, creating a sensitivity population of 207, while retaining the full data set for descriptive analyses and transparency.

Across both the unfiltered and filtered analyses, higher-order solutions (K = 3 and K = 4) consistently produced small clusters enriched for extreme observations and/or clusters with

negative silhouette values, indicating overlap rather than separation. In contrast, the two-cluster solution remained stable, demonstrated balanced cluster sizes, acceptable internal separation, and was not dependent on the presence of extreme values.

Furthermore, phenotypic characterization revealed that the two-cluster model captured the dominant axis of baseline heterogeneity, primarily driven by BMI, serum uric acid, and sex distribution. Additional clusters did not introduce qualitatively new phenotypes but instead fragmented this primary structure.

Taken together, these findings indicate that while the population exhibits continuous variation and intermediate profiles, its baseline structure is most robustly and parsimoniously represented by two dominant phenotypic clusters. The final two-cluster model was therefore selected as the primary representation of baseline heterogeneity, with higher-order solutions retained solely for exploratory sensitivity analyses.

Supplementary Table S2. Cluster-specific mean standardized values of K = 2 solution.

Variable	Adipose-metabolic (n = 116)	Lean–metabolic (n = 91)
**Age**	-0.011	0.014
**Diabetes duration**	0.031	-0.039
**BMI**	0.140	-0.178
**sUA**	0.332	-0.423
**HbA1c**	-0.048	0.061
**eGFR**	0.003	-0.004
**Sex**	0.884	-1.126

*

Supplementary Material S3. Evaluation of the three-cluster solution in the filtered cohorts.

The K = 3 solution yielded clusters of 54, 112, and 41 patients, and the separation remained primarily driven by BMI, sUA, and sex distribution, consistent with the K = 2 model. Two of the three clusters closely resembled the phenotypic extremes observed in the final K = 2 solution. One cluster was characterized by higher BMI and higher sUA levels with a predominance of male patients, whereas another cluster exhibited lower BMI and lower sUA with a predominance of female patients.

The third cluster displayed an intermediate phenotypic profile, with BMI and sUA values falling between those of the two dominant clusters. Sex distribution in this cluster was more balanced, and no single variable showed extreme deviation from the cohort mean. This cluster did not demonstrate clear separation from the adjacent clusters, as reflected by its lower and, in some cases, negative silhouette value (–0.186), indicating substantial overlap in multidimensional feature space.

Visualization using t-SNE further supported the interpretation that the three-cluster solution represented a continuum rather than discrete phenotypes, with the intermediate cluster occupying a bridging position between the two dominant metabolic profiles.

Taken together, the K = 3 solution suggests the presence of a gradual transition between two major baseline phenotypes rather than the emergence of a clearly distinct third subgroup. Accordingly, this solution was interpreted as exploratory, providing additional insight into the continuous nature of baseline heterogeneity but not offering a more stable or clinically actionable representation than the two-cluster model.

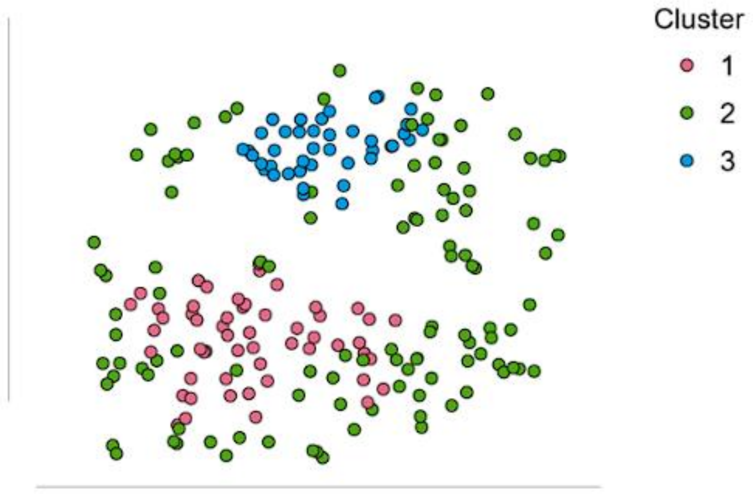

Supplementary Material S4. Evaluation of the four-cluster solution in the filtered cohorts.

The K = 4 solution resulted in cluster sizes of 43, 103, 15, and 46 patients and meant a further subdivision of the population along the same dominant metabolic axes identified in the two- and three-cluster models. Cluster separation continued to be driven primarily by BMI, sUA, and sex distribution; however, the additional partitioning did not reveal qualitatively new phenotypic patterns.

Two clusters largely corresponded to the adipose–metabolic and lean–metabolic phenotypes observed in the final K = 2 solution. A third cluster exhibited intermediate values for these variables, closely resembling the transitional phenotype identified in the three-cluster model with a silhouette score of –0.250.

The fourth cluster was characterized by a markedly smaller sample size (n = 15, silhouette score 0.421) and demonstrated extreme standardized values. Despite showing relatively high internal cohesion, this cluster contributed minimally to explaining overall population heterogeneity and was associated with reduced global separation metrics. This pattern indicates that the apparent separation of the fourth cluster was driven by isolation of a small subset of extreme observations rather than by the identification of a stable, generalizable phenotype.

Visualization using t-SNE confirmed substantial overlap among clusters, particularly between the intermediate and dominant phenotypes, further supporting the absence of clear boundaries in the four-cluster solution.

Overall, the filtered K = 4 solution did not improve phenotypic resolution compared with lower-order models. Instead, it fragmented the dominant metabolic structure into smaller components without enhancing interpretability or robustness. Accordingly, this solution was interpreted as indicative of overpartitioning and was not considered an appropriate representation of baseline population structure.

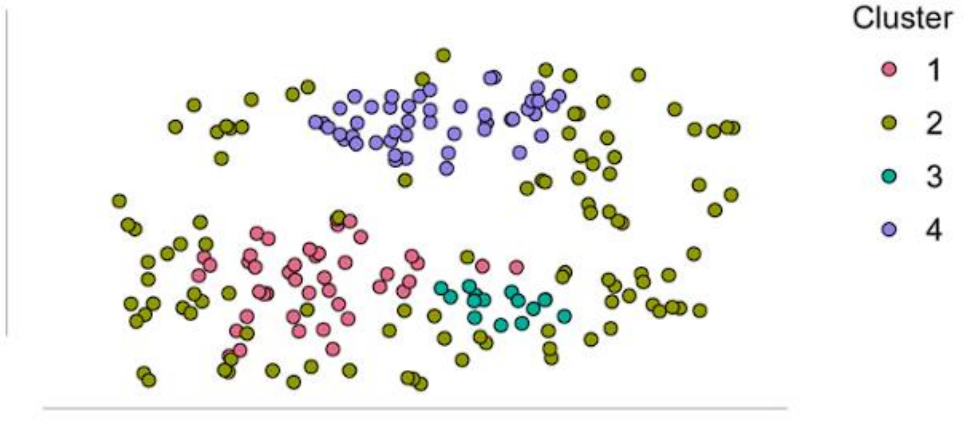

Supplementary Material S5. Detailed validation of cluster analysis.

To provide a quantitative assessment of internal cluster coherence beyond silhouette analysis, within-cluster variability was explicitly compared with total cohort variability for the primary clustering variables.

For each variable, SDs were calculated separately within each cluster and across the entire sensitivity cohort (n = 207). Cluster sizes were $n_1 = 116$ and $n_2 = 91$. To summarize within-cluster variability across clusters, a pooled within-cluster SD was calculated using the following formula:

$$SD_{\text{within}} = \sqrt{\frac{(n_1 - 1) \cdot SD_1^2 + (n_2 - 1) \cdot SD_2^2}{n_1 + n_2 - 2}}$$

The pooled within-cluster SD was then compared with the total cohort SD (SD_{total}) by computing the ratio:

$$R = \frac{SD_{\text{within}}}{SD_{\text{total}}}$$

Values of $R < 1$ indicate that variability within clusters is lower than variability in the overall cohort, consistent with meaningful internal cluster coherence. The variance-based validation was intentionally restricted to BMI and sUA, as these variables were the primary drivers of cluster separation and therefore represent the most clinically relevant dimensions for assessing internal cluster coherence.

Body mass index (BMI)

The observed standard deviations were $SD_1 = 5.65$, $SD_2 = 5.99$, and $SD_{\text{total}} = 5.86$.

Using the formulas above:

$$SD_{\text{within,BMI}} = \sqrt{\frac{115 \cdot 5.647^2 + 90 \cdot 5.991^2}{205}} \approx 5.80 \qquad \boldsymbol{R}_{\textbf{BMI}} = \frac{\mathbf{5.80}}{\mathbf{5.86}} \approx \mathbf{0.99}$$

Serum uric acid (sUA)

The observed standard deviations were $\mathrm{S}D_1 = 62.10, SD_2 = 67.04$, and $SD_{\text{total}} = 69.22$.

Using the formulas above:

$$SD_{\text{within,sUA}} = \sqrt{\frac{115 \cdot 62.10^2 + 90 \cdot 67.04^2}{205}} \approx 64.3 \qquad \boldsymbol{R}_{\textbf{sUA}} = \frac{\mathbf{64.3}}{\mathbf{69.22}} \approx \mathbf{0.93}$$

Both pooled within-cluster SDs were lower than the total cohort SD, indicating that patients within clusters were more homogeneous than the overall population. While the reduction in variability was modest, these findings support the presence of a structured, non-random clustering pattern rather than an artifact of arbitrary partitioning.

Supplementary Material S6. Sensitivity analysis excluding sex from clustering variables.

To evaluate whether the complete sex separation observed in the primary clustering solution influenced the identified phenotypic structure, a sensitivity analysis was performed in which sex was excluded from the set of clustering variables. Model-based clustering was repeated using the same baseline clinical parameters as in the main analysis.

Cluster sizes were comparable (n = 82 and n = 125), and no alternative solutions with higher cluster numbers demonstrated improved stability or interpretability. Quantitative validation metrics indicated weak cluster separation, with an overall silhouette score of 0.120 and cluster-specific silhouette values of 0.136 and 0.112, respectively. The proportion of explained variance was low ($R^2 = 0.117$), and within-cluster heterogeneity remained substantial in both clusters.

Visualization using t-SNE demonstrated partial visual separation between clusters; however, marked overlap persisted across the latent space, consistent with the low silhouette values and high within-cluster variance. Importantly, exclusion of sex did not lead to the emergence of more distinct or clinically interpretable metabolic phenotypes, nor did it materially improve cluster compactness or separation compared with the primary analysis.

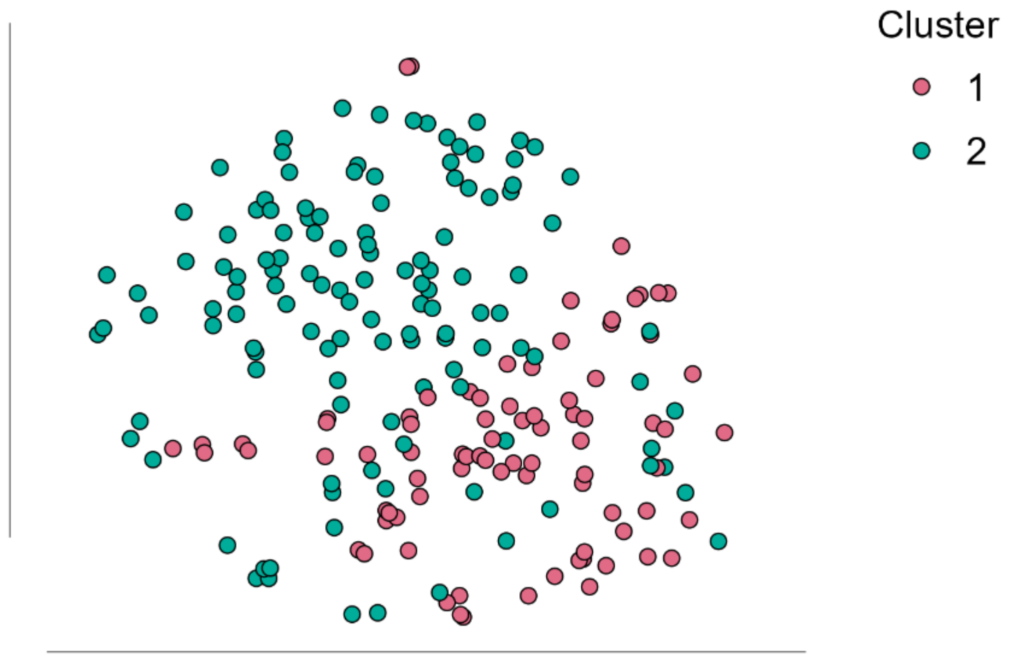

Supplementary Material S7. Baseline treatment allocation according to cluster-derived phenotypes.

Baseline treatment allocation did not differ between cluster-derived phenotypes ($\chi^2 = 0.929$, $df = 2$, $p = 0.629$; *Cramér's* $V = 0.067$), indicating comparable exposure to dapagliflozin, empagliflozin, and allopurinol across groups.

**Treatment**	**Adipose–metabolic (n = 116)**	**Lean–metabolic (n = 91)**	***p* value**
**Dapagliflozin (*n*, %)**	47 (40.5%)	31 (34.1%)	
**Empagliflozin (*n*, %)**	37 (31.9%)	33 (36.3%)	
**Allopurinol (*n*, %)**	32 (27.6%)	27 (29.7%)	
**Overall**			0.629
